# Polysaccharide-Based Micelles for Drug Delivery

**DOI:** 10.3390/pharmaceutics5020329

**Published:** 2013-05-27

**Authors:** Nan Zhang, Patricia R. Wardwell, Rebecca A. Bader

**Affiliations:** Syracuse Biomaterial Institute, 318 Bowne Hall, Syracuse University, Syracuse, NY 13244, USA; E-Mails: nzhang04@syr.edu (N.Z.); prwardwe@syr.edu (P.R.W.)

**Keywords:** polysaccharides, micelles, drug delivery, self-assembly, nanocarrier

## Abstract

Delivery of hydrophobic molecules and proteins has been an issue due to poor bioavailability following administration. Thus, micelle carrier systems are being investigated to improve drug solubility and stability. Due to problems with toxicity and immunogenicity, natural polysaccharides are being explored as substitutes for synthetic polymers in the development of new micelle systems. By grafting hydrophobic moieties to the polysaccharide backbone, self-assembled micelles can be readily formed in aqueous solution. Many polysaccharides also possess inherent bioactivity that can facilitate mucoadhesion, enhanced targeting of specific tissues, and a reduction in the inflammatory response. Furthermore, the hydrophilic nature of some polysaccharides can be exploited to enhance circulatory stability. This review will highlight the advantages of polysaccharide use in the development of drug delivery systems and will provide an overview of the polysaccharide-based micelles that have been developed to date.

## 1. Introduction

Micelles are self-assembled, nanosized colloidal particles with a hydrophobic core and hydrophilic shell [1]. The specialized structure makes micelles suitable carriers for poorly water soluble drugs that account for approximately 25% of conventional, commercially available therapeutics and nearly 50% of candidates identified through screening techniques [2,3]. Insoluble drugs often are characterized by poor bioavailability and rapid clearance after administration, characteristics that are associated with low therapeutic efficacy and high toxicity [4]. Micelles have been under investigation the past two decades to solve these issues [5]. Drug solubility has been greatly improved because of the hydrophilic shell of the micelle; and due to the tunable size of micelles, drugs can be directed to tissues where permeability is enhanced, particularly tumor and inflammatory tissue. Moreover, when modified by functional molecules that recognize molecular cues specific to diseased sites, micelles can achieve higher tissue specificity and cellular uptake [6,7]. To date, numerous micelle drug delivery systems have been developed, with some achieving clinical testing. However, some concerns, including material toxicity, immunogenicity, low cellular uptake, short half-life, and tissue accumulation, have arisen [8].

Early clinical results suggest that micelles should be designed and developed with careful attention towards material selection. Ideally, micelles developed for drug delivery should be biodegradable and should have high stability, high biocompatibility, and low immunogenicity. Natural polysaccharides meet the latter requirements and can be used to develop micelles in lieu of synthetic polymers. In addition, polysaccharides can be readily modified and exist in positive, negative, or neutral charge states. Finally, some polysaccharides are bioactive and can be used to augment the therapeutic efficacy of an associated drug or can enhance the targeting ability of a carrier system [9,10]. Despite these advantages, polysaccharide-based micelle systems are still under development and the outcomes have not met the clinical need. In this review, we will: (1) provide a more detailed depiction of the advantages offered by polysaccharides; (2) discuss the progress that has been made towards the application of polysaccharide-based micelles to drug delivery; and (3) offer suggestions for future research to expedite translation of polysaccharide-based drug delivery systems from the laboratory to a clinically relevant setting.

## 2. Advantages of Polysaccharide-Based Materials in Drug Delivery

Polysaccharides are a diverse class of polymeric materials of natural (animal, plant, algal) origin formed via glycosidic linkages of monosaccharides [11]. Dependent upon the nature of the monosaccharide unit, polysaccharides can have a linear or branched architecture. In addition to structural diversity, polysaccharides have a number of reactive groups, including hydroxyl, amino, and carboxylic acid groups, indicating the possibility for chemical modification [12]. Moreover, polysaccharide molecular weight can vary between hundreds and thousands of Daltons, further increasing diversity [13]. Herein, we will describe characteristics, including biocompatibility, solubility, potential for modification, and innate bioactivity, of several polysaccharides that lend credence to their potential for use in drug delivery systems.

### 2.1. Biodegradability and Biocompatibility

In contrast to many synthetic polymers, polysaccharides have very low (if any) toxicity levels [14,15,16,17]. For example, dextrans are biopolymers composed of glucose with α-1,6 linkages, with possible branching from α-1,2, α-1,3, and α-1,4 linkages, that exhibit low toxicity and high biocompatibility. Consequently, dextrans have formed the basis of biocompatible hydrogels for controlled prolonged therapeutic release [18]. Likewise, dextran has exhibited biocompatibility when formulated into microspheres, as suggested by a lack of an inflammatory response following subcutaneous injection into rats [19].

Also owing to their native presence within the body, most polysaccharides are subject to enzymatic degradation. Through enzyme catalysis, polysaccharides can be broken down to their monomer or oligomer building blocks and recycled for use as storage, structural support, or even cell signaling applications [20]. For example, glycosidases are common, constituting 1%–3% of the human genome [21], and can readily catalyze the hydrolysis of many different glycosidic linkages [20]. In contrast to glycosidase, other enzymes are more polysaccharide specific. Hyaluronidase, for instance, specifically degrades the polysaccharide hyaluronic acid (HA) by cleaving β-1,4 linkages between d-glucuronic acid and d-*N*-acetylglucosamine, particularly in regions of high HA concentration [20]. Of note, some polysaccharides are particularly susceptible to degradation by lysosomal enzymes, including glycosidases, esterases, and proteases, following endocytosis [22]. For example, lysozyme, *N*-acetyl-β-d-glucosaminidase, and a range of proteases play a role in the degradation of chitosan [23,24]. Thus, enzymatic degradation provides a mechanism of release for therapeutics associated with polysaccharide-based carrier systems [22].

### 2.2. Solubility

The functional groups along polysaccharide backbones, particularly hydroxyl and, to a lesser extent, amine groups, typically yield high aqueous solubility. However, this solubility can often be adjusted via monomer modification. For example, chitosan, composed of β-1,4 linked *N*-acetyl-d-glucosamine and d-glucosamine, is prepared via deacetylation of chitin. By varying the degree of deacetylation of the parent compound, the solubility of chitosan in acidic conditions can be tuned. Higher degrees of deacetylation correspond to an increased number of available, protonated free amino groups along the polysaccharide backbone and, consequently, enhanced solubility [25]. Likewise, *O*-acetylation of glucomannan, a polysaccharide formed via β-1,4 linkage of d-mannose and d-glucose, can be used to modulate the formation of intermolecular hydrogen bonds with water, thereby altering aqueous solubility [26].

### 2.3. Ease of Modification

Polysaccharides are extremely amenable to modification. For example, glucose-based polysaccharides, such as amylose, amylopectin, glycogen, and cellulose, offer an abundance of free reactive hydroxyl groups [8]. Other polysaccharides possess both hydroxyl and carboxylic acid moieties that can be readily modified. For example, a review was recently published focusing on the derivatization of alginate, a polysaccharide composed of β-d-mannuronic acid and α-l-guluronic acid with 1,4 linkages. Alginate modification can be used to give rise to a variety of different physiological behaviors. For instance, hydroxyl group oxidation enhances biodegradability, while sulfonation generates a heparin-like polysaccharide with increased blood compatibility (see Section 2.4 for additional details on heparin) [9]. Modification of chitosan has also been extensively reviewed. Specifically, quaternization of the primary amines with various alkyl groups can be used to enhance solubility and alter bioactivity [27,28,29].

### 2.4. Bioactivity

Many polysaccharides possess innate bioactivity, particularly mucoadhesive, antimicrobial, and anti-inflammatory properties. Mucoadhesion refers to the interaction of a material with a mucosal layer, such as in the gastrointestinal (GI) tract, nasal pathway, or airway. Chitosan, the only natural, positively charged polysaccharide, is capable of binding to the negatively charged mucosal layers through charge interactions [30,31,32]. Thus, numerous investigators have explored the use of chitosan for oral drug delivery. For neutral or negatively charged polysaccharides, such as HA, hydrogen bonding provides an alternative mechanism for mucoadhesion [33]. Several polysaccharides are also antimicrobial in nature. The cidal effects of chitosan, for example, are presumed to be due to a strong interaction of the protonated amines with the negatively charged bacterial cell wall [34]. Other polysaccharides are known to reduce inflammation. For instance, heparin, which is composed of repeating disaccharides of β-D-glucopyranosiduronic acid or α-L-idopyranosiduronic acid linked to *N*-acetyl or *N*-sulfo-D-glucosamine, has the strongest negative charge of any polysaccharide, which enables interaction with a variety of proteins. Thus, anti-inflammatory activity is thought to be due to binding with immune-related acute phase and complement proteins [31,35]. In addition, heparin can bind to the lysine-rich region of antithrombin, thereby catalyzing the inhibition of blood clotting [36,37].

## 3. Polysaccharide-Based Micelle Drug Delivery Systems

**Figure 1:**
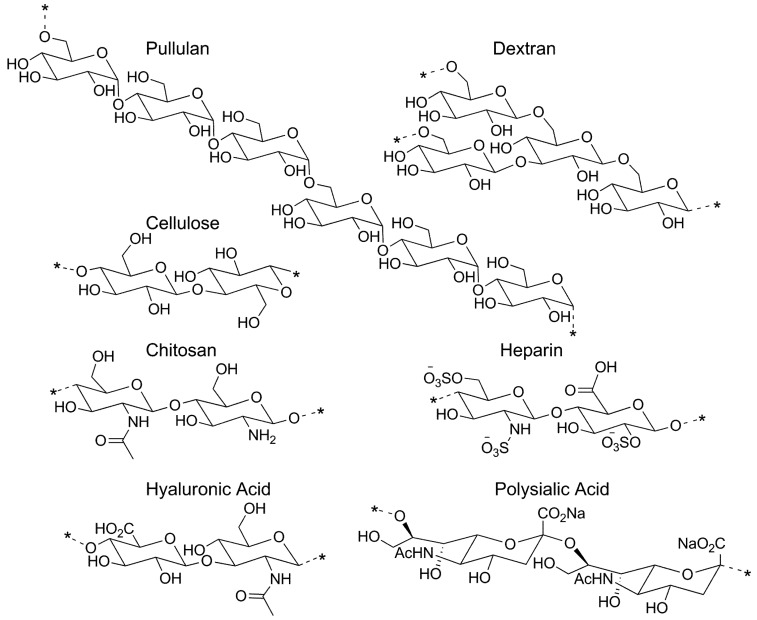
Structures of polysaccharides that are used in the development of micelle drug delivery systems.

A number of polysaccharides have been used to date in the development of micelles for drug delivery. The structures of the more commonly used polysaccharides are given in Figure 1. The numerous functionalities along the polysaccharide backbone facilitate attachment of a hydrophobic moiety that can be used to initiate self-assembly to a micelle. The most prevalent hydrophobic groups used in micelle formation are provided in Table 1, along with example references. Of note, several investigators, particularly those that study cholesterol modified pullulan [38,39,40], have reported the formation of multiple hydrophobic microdomains, rather than a single hydrophobic core, upon self-assembly of the hydrophobized polysaccharides. These microdomains serve as physical crosslinks within the confines of the exterior hydrophilic shell, and consequently, the self-assembled constructs formed in this manner can be referred to as nanogels [41,42]. The nature of the hydrophobic core (single versus multiple hydrophobic domains) is dependent upon the concentration of the amphiphilic polysaccharide [43], the nature of the hydrophobic group used for polysaccharide modification [44], and the degree of substitution of the hydrophobic group along the polysaccharide backbone [43,44]. For simplicity, all systems generated via self-assembly of hydrophobically modified polysaccharides will be referred to as micelles in this review.

### 3.1. Pullulan-Based Systems

Pullulan is a water-soluble, neutral, non-toxic bacterial exopolysaccharides [45]. Since Akiyoshi *et al*. first reported cholesterol-bearing pullulan (CHP) as a self-aggregated colloidal system with high stability [41,46], numerous studies related to or based on CHP have been carried out. In the original report, CHP was synthesized by grafting 1.6 cholesterol groups to every 100 glucose units on pullulan (M_w_ 55,000 g/mol, M_w_/M_n_ = 1.54) in a random manner. The average hydrodynamic radius of CHP self-aggregates was measured by dynamic light scattering (DLS) and determined to be 13.3 nm. Morphology studies by negatively stained transmission electron microscopy (TEM) confirmed that CHP can self-aggregate as spherical particles with relatively uniform size. Further studies via size exclusion chromatography (SEC) and ^1^H-NMR provided information that one CHP self-aggregate was composed of approximately 13 CHP molecules and that these molecules formed a rigid hydrophobic cholesterol core with a relatively mobile pullulan shell. More importantly, CHP self-aggregates revealed very high colloidal stability [40] and have since been investigated as a way of protecting proteins from the physiological environment. Studies have shown that CHP self-aggregates can be loaded with insulin with high binding constant (*K* = 2 × 10^6^ M), thereby protecting the entrapped protein from thermal denaturation and enzymatic degradation [47,48].

**Table 1:** Names and structures of hydrophobic moieties used in the development of polysaccharide-based micelle drug delivery systems. Functional groups used for grafting onto polysaccharides are highlighted in red.

Name	Structure	References
Cholesterol	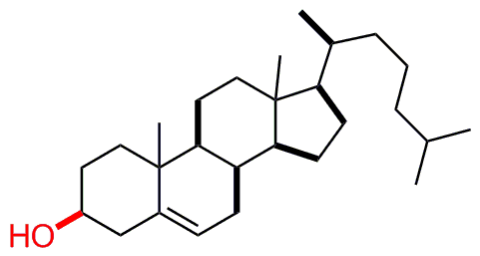	[38,39,40,46,49,50,51,52,53,54,55,56,57]
Cholic Acid	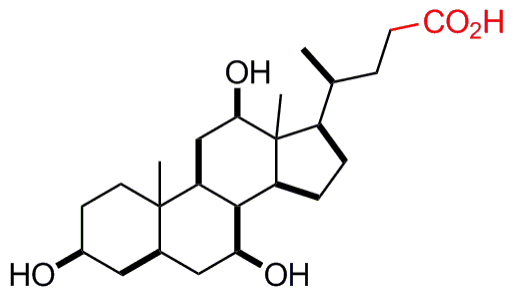	[43,58,59,60]
Deoxycholic Acid	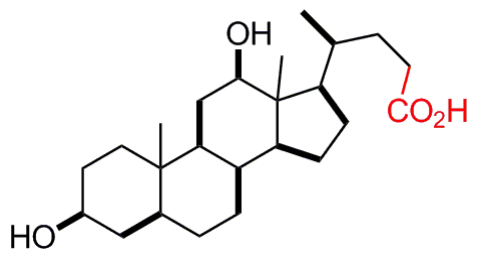	[43,44,58,61,62,63,64,65,66,67,68,69,70]
Poly(lactide)	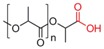	[71,72,73,74,75,76]
Poly(lactide-co-glycolide)	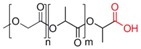	[55,77,78,79,80,81,82]
Pluronic	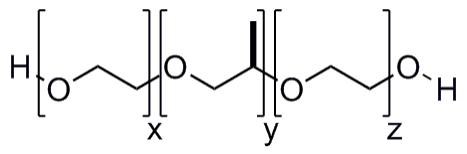	[83,84,85,86,87,88,89,90,91,92,93]
Polycaprolactone	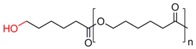	[93,94,95,96,97,98,99,100]
Stearic Acid	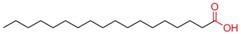	[58,79,101,102,103,104,105,106,107,108,109,110,111]

More recently; CHP has been further modified to form more complex drug delivery systems for higher demand. For example; CHP cross-linked hydrogels with thiol-bearing PEG [57] and CHP cross-linked membranes (diameter 6 mm; thickness 0.4 mm) [53] have been used for critical bone defect treatment. Results showed that the crosslinked CHP membrane could stimulate and promote bone regeneration with an improved outcome relative to a control; collagen membrane [53]. Moreover; the CHP crosslinked hydrogel was successfully used to co-deliver recombinant human bone morphogenetic protein 2 and fibroblast growth factor 18 (FGF 18) to induce enhanced bone repair [57]. CHP has also be used to deliver vaccines [52]; anti-tumor agents [50,51]; and wound healing agents with improved solubility and stability. Of note; blank CHP; *i.e.*, CHP used in the absence of additional therapeutics; showed a positive effect on wound healing compared to control [49].

Other pullulan based-micelle systems include pullulan acetate [112], poly(DL-lactide-co-glycolide)-graft pullulan [77], pullulan-*g*-poly(L-lactide) [71,76], and pullulan hydrophobic drug conjugates, such as pullulan-doxorubicin (DOX) [113] and pullulan-biotin [114]. These systems were mainly investigated in regards to chemical synthesis, physicochemical characterization, and drug release. To elucidate the potential of these systems for drug delivery, additional studies should focus on the interaction of the micelles with cells, tissues, and living systems.

### 3.2. Cellulose-Based Systems

Cellulose is the most abundant naturally occurring polysaccharides and its derivatives have been widely used in the pharmaceutical field. For example, hydroxypropyl cellulose (HPC) was produced by modifying some of the cellulose hydroxyl groups with propylene oxide to improve the cellulose solubility and control drug release [115]. To improve the oral delivery of hydrophobic drugs, which often have poor bioavailability after administration, Winnik *et al*. further modified HPC with hydrophobic hexadecyl (C_16_) or octadecyl (C_18_) groups through a short, polyoxyethylene (POE) linker of variable length [HPC-(POE)_10 or 20_-C_16 or 18_]. With five hydrophobic molecules per HPC chain, the critical micelle concentration (CMC) was 65–135 mg/L while with ten hydrophobic chains, the CMC dropped to 15–22 mg/L. Cyclosporin A (CyA), a poor water-soluble immunosuppressant, was used as a model drug in this study. With lower hydrophobic modification, the maximum loading was 0.025 mg CyA/mg micelle, while, as anticipated, with higher modification, the loading capacity increased to 0.067 mg CyA/mg micelle. With the same quantity of hydrophobic moieties, PEO-C_16_ provided an improved solubilizing environment for CyA relative to PEO-C_18_. With the encapsulation of CyA, the polymeric micelles size dropped from 78–90 nm to 44–74 nm. Presumably, the encapsulation of CyA enhances the hydrophobic interactions in the core and produces more compact particles. *In vitro* studies showed HPC-PEO-C_16_ micellar system had high affinity to mucus and could enhance the permeability of entrapped therapeutics across intestine epithelial-Caco-2 cells [60,116,117,118,119,120,121,122,123,124,125,126,127]. These studies demonstrated the great potential of HPC-based micelles for improved oral delivery of hydrophobic molecules.

Other work has focused on the design and synthesis of cellulose-based micelles; however, how these systems can be applied to the field of drug delivery has not yet been described. Such systems include HPC-polycaprolactone [98] and cellulose-C_15_-pyrene micelle. Of interest, micelles prepared from cellulose-C_15_-pyrene with longer cellulose chains (M_w_ = 4860 g/mol, number average degree of polymerization (D_n_) = 30) were smaller in size (~40.0 nm, monolayer micelle) relative to those prepared from short chain cellulose (M_w_ = 2106 g/mol, Dn = 13) (~108.8 nm, multilayer micelle) [119].

### 3.3. Dextran-Based Systems

Dextran is another polysaccharide that has long been used in drug formulation and has shown no toxicity [120]. For this reason, Winnik *et al*. conducted a series of studies on dextran-based micelles for CyA oral delivery that were nearly identical to the experiments conducted for modified CHP. Similar to HPC-PEO-C_16_, they demonstrated dextran-PEG-C_16_ had a higher loading capacity of CyA relative to dextran-PEG-C_18_ (0.048 *vs*. 0.03 mg CyA/mg micelle), although the dextran-based systems yielded higher loading capacities overall compared with the HPC-based systems. The size of dextran-PEG-C_16_ was very small (9 ± 0.3 nm), and CyA loading did not significantly affect micelle size (10 ± 0.3 nm). Although not yet tested, this small size may limit future in vivo applications. Dextran-PEG-C_16 _showed no toxicity to Caco-2 cells after 4 h of exposure, although free PEG-C_16_ did inhibit cell growth [121]. Additional *in vitro* studies demonstrated that dextran-PEO-C_16_ could significantly improved CyA permeability across Caco-2 cells, although the improvement was lower than that achieved by CyA loaded HPC-PEO-C_16_ [119] and, unlike HPC-PEO-C_16_, dextran-PEO-C_16_ showed no affinity to mucus [118]. To improve the relative low transport efficiency, vitamin B_12_ was conjugated to the micelle and the vitamin B_12_-dextran-PEO-C_16_ showed increased transportation of CyA across the Caco-2 monolayer and internalization of CyA by Caco-2 cells via the vitamin B_12_ pathway [122].

Another highly investigated system is dextran-cholic acid. Cholic acid is one of the major bile acids that help to deliver and digest hydrophobic fats in the human small intestine via bile acid self-aggregates. Early dextran-cholic acid systems had low stability, as indicated by a high CMC value (0.02–0.2 g/mL) [44]. The CMC can be described by *C*_CMC_ ~ exp(−nε_h_/*k*_b_*T*), where *k_b_T* is the thermal energy and ε is the effective interaction energy between the monomer and the bulk solution. Therefore, a high CMC, as obtained with dextran-cholic acid, is suggestive of low thermodynamic stability [128]. Improved systems were developed by Yuan *et al*. [124] and Xu *et al*. [125] based on periodate-oxidized dextran which possessed free aldehyde and hydroxyl group that were used to form hydrogen bonds to increase system stability. Moreover, the micelles served as good depots for the hydrophobic drug indomethacin (~0.299 mg drug/mg micelles) and showed sustained release up to 14 days at acidic and neutral condition. More recently, dextran sulfate-cholic acid was investigated to deliver superoxide dismutase (SOD) orally. SOD-loaded dextran sulfate-cholic acid had a high stability against the acidic environment of the stomach and release in the small intestine was controlled up to 100 h. Moreover, dextran sulfate-cholic acid facilitated SOD cellular uptake, suggesting that cholic acid enhanced the interaction of micelles with the intestinal membrane [60]. Additional dextran-based micelles have been synthesized by grafting of polycaprolactone [95], poly (L-lactide) [55], polystyrene [126], lauryl group [127], and methyl methacrylate-ethylene glycol dimethacrylate [128] to the dextran backbone.

### 3.4. Chitosan-Based Systems

Chitosan and its derivatives have been the most widely investigated material for drug delivery due to the superior properties. To improve delivery of hydrophobic molecules, many studies have focused on chitosan-based micelle systems. Most of these core-shell systems were developed by modifying chitosan with hydrophobic moieties that include stearic acid [106,107,108,109,110], (deoxy)cholic acid [58,66,67,68], glycyrrhetinic acid [58,129], polycaprolactone [93,94], *etc*. Self-assembly of the modified chitosan can lead to the formation of spherical micelles with a size range of 20–500 nm in aqueous solution. Higher hydrophobic moieties modification percentage usually gives rise to a smaller micelle diameter due to stronger hydrophobic interactions. Various anti-tumor therapeutics such as paclitaxel (PTX) [94,101,130,131], doxorubicin [58,65,66,92,132,133], and camptothecin [134], have been used as model drugs and encapsulated by chitosan-based micelles. The chitosan-based micelles improved the solubility of the hydrophobic drugs significantly. Moreover, the micelles showed controlled or sustained release of the hydrophobic drugs, and the release rate was tunable by the degree of substitution (DS) of the hydrophobic moieties of the micelles. Higher DS usually indicated slower drug release despite insignificant changes in the loading efficiency. The therapeutic-loaded micelles showed significantly higher toxicity to tumor cells *in vitro* compared to free drugs due improved drugs internalization.

Due to the muco/bioadhesive nature of chitosan (Section 2.4), chitosan-based micelles have been used extensively to improve the oral drug delivery. Evaluated by a Caco-2 cell monolayer, chitosan-based micelles were demonstrated to inhibit the activity of P-glycoprotein 1 (P-gp) ATPase, which, consequently, can inhibit drug efflux and enhance drug permeation [105,132]. Moreover, the chitosan opened the tight junctions between cells and further enhanced drug absorption. The chitosan-based micelles were characterized by low CMCs, suggestive of high stability [135] and resistance to the harsh environment of the GI tract. *In vivo* studies showed that *N*-octyl-*O*-sulfate chitosan can improve the oral bioavailability of PTX by 6 folds compared to the current commercially improved formulation-Taxol (Bristle-Myers Squibb, New York, NY, USA) [132]. Additionally, chitosan-based micelles were demonstrated to be a relatively safe carrier for oral formulation [136]. Chitosan-based micelle systems have also been investigated for applications in antivirus [103], anti-thrombogenicity [137], and antiplatelet aggregation [135].

### 3.5. Heparin-Based Micelle Systems

Heparin is a polysaccharide that has a variety of biological functions, such as anticoagulant activity, inhibition of angiogenesis and anti-tumor development [138,139]. Therefore, several studies have focused on heparin-based micelle systems for improved cancer treatment. Pluronic block copolymer, composed of hydrophilic poly (ethylene oxide) (PEO) and hydrophobic poly (propylene oxide) (PPO) in triblock structure: PEO-PPO-PEO, can improve oral availability of hydrophobic drugs by increasing the solubility and permeability while inhibiting the activity of P-gp mediated drug efflux and cytochrome P450 metabolism [85,86]. To develop an oral anti-tumor formulation, heparin-pluronic micelles were developed to improve drug absorption. These systems showed proper diameter for tumor accumulation and high loading efficiency for PTX and RNase A, an anti-tumor protein. The therapeutic-loaded heparin-pluronic micelles showed 5–6 folds higher permeability through rat intestines relative to Taxol [83,84]. Oral availability was also improved by grafting deoxycholic acic to low molecular weight heparin. Deoxycholic acid-heparin micelles possessed a diameter of 100–200 nm and were absorbed in the small intestine via a bile acid transporter, as shown with a nude mouse model [61,62]. Other micelle systems include heparin-PTX [140,141] and heparin-poly(β-benzyl-l-aspartate) [142].

### 3.6. Hyaluronan-Based Systems

HA is bioactive in that it can bind to the CD44 receptor that is overexpressed in tumor and inflammatory tissues. Thus, HA has been investigated as an active targeting agent in drug delivery for enhanced efficacy. Most recent HA-based micelle systems have focused on cancer treatment with DOX [83,143,144,145], PTX [63,146,147], siRNA [148], and curcumin [149]. These systems possessed high physiological stability and a diameter range of 100–200, which is expected to facilitate passive accumulation of the micelles in the tumor. HA-based micelles showed significantly higher cellular uptake by a CD44 overexpressed cancer cell line compared to a CD44 negative cell line, NIH3T3 [143]. Moreover, by blocking the CD44 receptor with free HA molecules, cellular uptake of HA-based micelles was significantly decreased [63,82]. Therefore, HA-based micelle systems were demonstrated as potential system for improved drug efficacy via CD44-mediated endocytosis. To further improve targeting, folic acid, another active targeting agent was conjugated to HA and higher cellular uptake was observed with folic acid-HA-octadecyl group compare to HA-octadecyl group [146]. However, due to the high affinity of HA to liver sinusodidal endothelial cells that have another HA receptors (HARE), HA-based micelles have a high propensity for accumulation in the liver after systemic administration. In order to circumvent this, PEG was conjugated to HA-5 beta-cholanic acid and liver accumulation of micelles was significantly suppressed, while the tumor accumulation was increased to 1.6 folds. Intravital tumor imaging also confirmed PEG-HA-5 beta-cholanic acid had rapid extravasation into tumor tissue [150].

### 3.7. Other Polysaccharide-Based Micelle Systems

One important advantage of polysaccharides is their large diversity of biological functions (Section 2.4). Advances of polysaccharide research have provided more candidates as potentially functional biomaterials. In addition to the most investigated polysaccharides described above, several other polysaccharides have also been used to develop micelle systems. Polysialic acid (PSA), in particular, is a non-toxic polysaccharide that can be used to protect and increase body circulation of therapeutics and has been developed into micelle systems for the delivery to inflamed tissue. Using CyA as a model drug, a high loading capacity was achieved with micelles prepared from polycaprolactone (PCL) modified PSA [151]. Alginic acid-PEG showed very significant enhancement of hypocalcemia efficacy in rats after intraduodenal administration and can improve the oral absorption of salmon calcitionin via alginic acid-PEG facilitated transcytosis across Caco-2 cells [152]. Mannan based-micelle systems with high stability were developed by grafting cholesterol [153] or hexadecanethiol (C_16_) [154,155] to mannan. In addition, Modolon *et al*. have led an investigation of maltoheptaosyl-based micelles composed of hydrophilic maltoheptaosyl and hydrophobic peracetylated maltoheptaosyl [156]. The all polysaccharide micelle is expected to exhibit better properties and functions.

## 4. “Smart” Polysaccharide-Based Micelle Drug Delivery Systems

### 4.1. Stealth Coating

To achieve high therapeutic efficacy, therapeutics must have a long circulation time in the body to ensure that an effective concentration at the target site is achieved. However, free drugs and many carrier systems bind to plasma proteins and are rapidly cleared. To minimize clearance and prolong the circulation, PEG has been conjugated or coated to many micelle systems. For example, studies have shown that PEG protected octyl-succinyl-chitosan from plasma protein absorption [157] and that PEG modification could prolong HA-ceramide circulation [143]. Moreover, PEG could inhibit liver uptake of HA-conjugated micelles, thereby increasing the systemic circulation [150].

Despite the demonstrable improvements relative to unmodified systems, there are potential drawbacks of PEG usage, such as the non-biodegradable PEG backbone, continuous accumulation in the body, and possible induction of an immune response [158,159,160]. In addition, the PEG coating may interfere with cellular uptake of drugs because PEG has been reported to reduce drug-cell interaction and to hinder the drug release from carrier systems [161,162,163]. As an alternative, other molecules that are hydrophilic, biodegradable, non-toxic, and non-immunogenic have been sought. For example, polysialic acid (PSA) meets all of the latter criteria and, of equal importance, PSA has no known receptors in the human body, suggesting the possibility for further improvement in circulatory stability. Gregoriadis *et al*. have investigated a series of PSA-protein conjugate and shown prolonged circulation of insulin [164], asparaginase [165,166], and catalase [167,168]. Recently, Bader *et al*. synthesized PSA-based micelle systems for future applications in drug delivery [151].

### 4.2. Stimuli-Sensitive Systems

Stimuli-sensitive systems can provide more controllable drug delivery with better efficacy. Based on the solution to gel phase transition properties of polymer, thermo-sensitive systems have been developed. For example, Kim *et al*. reported an injectable, low molecular weight methylcellulose-pluronic gel/micelle system that was a solution at 25 °C and gel at physiological temperature. The solution phase is expected to facilitate injection, while the gel phase was demonstrated to sustain drug release for approximately 3 weeks [169]. Likewise, a pullulan-based micelle system contained thermo-sensitive poly(L-lactide) that gelled at 42 °C. Gelation induced additional anti-cancer drug release and, consequently, better inhibition of tumor cell growth [71].

pH-Sensitive system have also been developed. Several intracellular structures, particularly endosomes and lysosomes, are characterized by a low pH. In addition, tumor and inflamed tissue often possess a pH that is slightly lower than normal (6.5–7.2). Thus, by developing pH-sensitive systems, premature leakage of drugs from the micelles can be reduced and maximum drug release can be achieved at the target site. To date, almost all such systems have been developed for tumor treatment. Selective release at the tumor site is accomplished by incorporating various moieties with hydrophobic and electronic interactions that change with pH [66,146,157,170,171].

Chemical signals can also provide a method for site-specific drug release from micelles. For instance, glutathione (GSH) concentration in tumor cells is 4 folds higher than in mammalian cells and 3 orders higher than in plasma. The high concentration of GSH in tumor cells can attack the unsaturated and disulfide bonds that exist within macromolecules and facilitate degradation or other structural changes. Therefore, polysaccharide-based micelle systems that contain these sensitive bonds (so called “redox (reduction-oxidation)-sensitive systems”) have been investigated for tumor targeting and intracellular efficacy enhancement. As an example, a negligible amount of prodrug was released from carboxymethylchitosan-based micelles without GSH and at low concentrations of GSH; however, 75% of the conjugated drug was released with the presence of GSH at 20 mM [172,173]. Likewise, Li *et al.* led a study of redox-sensitive micelles, specifically HA-deoxycholic acid conjugates that were loaded with PTX for tumor targeting. At GSH 20 mM, the micelles underwent fast disassemble and released the PTX into the cancer cells. The redox-sensitive, PTX-loaded HA-based micelles showed higher tumor targeting capacity and more potent efficacy towards cancer cells compared to an insensitive control [63]. Heparin-pluronic-based micelles that are responsive to high GSH concentrations have also been developed [89].

Although not well explored, evidence suggests that photosensitive polysaccharide-based micelles may serve as functional, smart materials in drug delivery. Modification of pullulan with spiropyrane yielded hydrophobized polysaccharide that self-assembled into micelles. The amphiphilicity of the spiropyrane core was modulated through irradiation with visible light, which consequently impacted the interaction and release of associated proteins [174]. Similarly, release of model compounds from micelles formed from azobenzene-dextran was controlled through exposure to UV-Vis light [175].

### 4.3. Active Targeting Agents

Micelles with proper size, charge, and shape will facilitate improved delivery. However, this may not be enough because of the defenses within the human body. To enhance target site accumulation and uptake, additional targeting agents are often incorporated into the micelle systems. Active targeting agents used in polysaccharide-based micelles can be divided into three categories: peptides, small molecules, and polysaccharides. Peptides include octreotide targeting for the somatostatin receptors on tumor cells [65,157], the A54 hepatocarcinoma binding peptide [105], and Arg-Gly-Asp (RGD) containing peptide for α_v_β_3_ and α_v_β_5_ integrins [89,176]. Small molecules include glycyrrhetinic acid, a liver targeting ligand [58,139], vitamin E succinate-specific toxicity to tumor cells [131], and folic acid with a high affinity to the folate receptor overexpressed on tumor cells [64,101,146,177,178,179]. Through peptide and small molecule conjugation, micelle systems have shown higher tumor accumulation and cellular uptake relative to blank micelles. Moreover, polysaccharides themselves can act as active targeting agents due to their bioactivity. As described above, HA has a high affinity to the HA receptor on the liver sinusoidal endothelial cells and the CD44 receptor that is overexpressed on the tumor cells and inflamed synovial fibroblast. Therefore, HA has been highly investigated as an active targeting agent to tumor tissue or liver with many micelle systems [82,143,144]. In addition, heparin-based micelles have demonstrated enhanced inhibition of tumor growth and angiogenesis [138,139], while cellulose [116,117,118], chitosan [104,139], and pullulan-based [180] systems promoted drug absorption across the small intestine due to enhanced mucoadhesion.

## 5. Conclusions and Outlook

As drug carrier systems, polysaccharide-based micelle systems have shown potential to improve hydrophobic drug and protein delivery through enhanced solubility, increased stability, and controllable drug release properties. Moreover, cellulose and chitosan-based micelles showed improved oral absorption due to mucoadhesion and many polysaccharide-based micelles have demonstrated enhanced tumor penetration and inhibition. In the past two years, many in depth studies have been published on the investigation of polysaccharide-based micelles-cells interactions and the *in vivo* behavior of these micelles. Several trans-mucus mechanisms of micelles have been proposed and receptor mediated endocytosis has been demonstrated for many micelle-cell interactions. Polysaccharide-based micelles were shown with prolonged circulation and favorable pharmacokinetics in several mouse models, indicating the potential for translation to clinical research. From the progress of the polysaccharide-based micelles, there is a clear trend towards more complex and controllable systems, which will possess higher targeting and specificity to further improve therapeutic efficacy and reduce undesired side effects.
